# Apparent Pathologies in Stochastic Entropy Production in the Thermalisation of an Open Two-Level Quantum System

**DOI:** 10.3390/e28020196

**Published:** 2026-02-10

**Authors:** Jonathan Dexter, Ian J. Ford

**Affiliations:** Department of Physics and Astronomy, University College London, Gower Street, London WC1E 6BT, UK; jonathandexter84@gmail.com

**Keywords:** open quantum system, quantum thermodynamics, stochastic entropy production

## Abstract

We investigate the entropic consequences of the relaxation of an open two-level quantum system towards a thermalised statistical state, using a framework of quantum state diffusion with evolution described by a minimal set of raising and lowering Lindblad operators. We demonstrate that thermalisation is typically accompanied by a persistent non-zero mean rate of change of the environmental component of stochastic entropy production. This thermodynamic signature can be associated with the purification of the reduced density matrix ρ of the randomly evolving state under these dynamics, which may be contrasted with the impurity of the more frequently considered ensemble average of ρ. The system adopts stationary statistics, with zero stochastic entropy production, after a further stage of relaxation once purity has been achieved. We show that apparent pathological mathematical difficulties in the computation of stochastic entropy production emerge in this evolution towards stationarity if ρ is represented using a certain set of coordinates, though these problems can be removed by choosing a different set. We conclude that frameworks for modelling open systems must be carefully selected to provide satisfactory thermodynamic as well as dynamic behaviour.

## 1. Introduction

The second law of thermodynamics emerges when the world is modelled at a coarse-grained level but governed by underlying equations of motion with a sufficient degree of deterministic chaos. The increase in entropy corresponds to a growth in the inescapable subjective uncertainty regarding the adopted configuration of the world in such a situation, and this applies both to classical and to quantum systems [1,2,3]. To illustrate this, we study the evolution of an open two-level quantum system using a framework of stochastic dynamics that represents the effects of coupling to an underspecified environment. We treat the reduced density matrix of the system as a set of physical coordinates that undergo Brownian motion, and concern ourselves with the mechanical and thermodynamic approach towards a thermalised state. The stochastic entropy production that accompanies the evolution is a measure of its thermodynamic irreversibility and identifies an arrow of time [4].

We employ a framework of quantum state diffusion [5] where the system dynamics are modelled using Itô processes, Markovian stochastic differential equations (SDEs) with continuous solutions. The associated stochastic entropy production can also be described using an SDE [6]. We find several apparently pathological features in the stochastic entropy production. In Section 3, we describe persistent stochastic entropy production accompanying the thermalisation of the system by the environment. We associate this behaviour with asymptotic purification of the state under the chosen dynamics such that a stationary statistical state is reached only in infinite time. Furthermore, the relaxation of a purified system towards the stationary thermalised state can appear to be burdened with mathematical difficulties in the form of singularities in stochastic entropy production. We investigate and resolve such issues in Section 4.

Our conclusions are given in Section 5. Appendix A provides a discussion of the basis for computing stochastic entropy production, and Appendix B is a summary of how this may be achieved in situations characterised by restricted diffusion [7]. Finally, Appendix C explores some unexpected mathematical features that arise in the averaging of SDEs, and how they can lead to additional terms in the mean system component of stochastic entropy production.

## 2. Quantum Trajectories of the Reduced Density Matrix

We first review the picture that underpins our interest in the evolution of a reduced density matrix along a stochastic trajectory. In the absence of projective quantum measurement, the full density matrix of a system together with its environment (a closed ‘world’) evolves deterministically according to the unitary dynamics of the Schrödinger equation. On the other hand, the reduced density matrix, which is the part of the full density matrix that represents properties of the system and is often described as the result of ‘tracing out’ the environment, evolves with the preservation of unit trace and positivity but with the potential for changes to von Neumann entropy and purity, effects that emerge from thermalisation, for example [8,9,10].

However, the trajectory followed by the reduced density matrix will be unpredictable if the complete initial state of the world is not specified in the model. Assuming that the dynamics are suitably chaotic, it might be possible to regard the evolution as an effective Brownian motion of the reduced density matrix driven by noise of some kind, noting, however, that the dynamics would be pseudorandom rather than intrinsically stochastic.

Averaging over many such Brownian trajectories would represent the statistically expected behaviour of a system coupled to an underspecified environment, and this would emerge from use of the usual non-stochastic Lindblad equation [9,10]. But we regard the actual behaviour of the system to be represented by the individual trajectories, rather than their average [2,3]. If the state vector of the system and environment is ontic, the trajectories of the reduced density matrix are a closer reflection of reality than the more frequently considered mean behaviour. The stochastic entropy production that we compute in this study is a measure of the mechanical and thermodynamic irreversibility of this pseudorandom evolution.

It is worth contrasting this with the idea of false decoherence [11]. From our point of view, such a transformation of the reduced density matrix from one that is pure to one that is mixed refers to the evolution of the average over an ensemble of possible trajectories of the system rather than the behaviour corresponding to an individual trajectory. The lack of control over the environmental interaction produces an ensemble of individual system states with uncertain relative coherence. The ensemble average is a reduced density matrix that is not pure (i.e., it is mixed), and this is false decoherence. True decoherence is an entanglement with the environment that can reduce the purity of the reduced density matrix. In contrast, environmental interactions in our model of individual state evolution can potentially purify the system state, if it were initially entangled with the environment. In our framework a specific form of environmental interaction can bring about measurement of the system, for which purification is necessary and natural since it has to evolve the system to an eigenstate of the measured operator [2,3].

Note that the von Neumann entropy refers to the uncertainty of projective measurement outcomes for a set of basis states in which the reduced density matrix is diagonal. It differs from the stochastic entropy production computed here, which is associated with the change in subjective uncertainty in the quantum state vector of the world.

## 3. Thermalising a Two-Level Open Quantum System

### 3.1. System Dynamics

A stochastic Lindblad equation for the evolution of the reduced density matrix ρ of an open system can be constructed using Lindblad operators that describe the influence of the environment on the system [2,3,9,10,12]. Such an approach allows us to model the evolution of ρ given an underspecified state of the environment: a concept familiar in classical statistical mechanics and also applicable in quantum mechanics. No projective measurement is imposed. Environmental interactions drive quantum state diffusion where ρ follows a continuous Brownian trajectory [5].

We consider a two-level quantum system with environmental couplings characterised by the raising and lowering Lindblad operators given by

(1) $$c_{+}=\left(\begin{array}{cc} 0 & 0\\ 1 & 0 \end{array}\right),\;c_{-}=\left(\begin{array}{cc} 0 & 1\\ 0 & 0 \end{array}\right),$$

expressed in a basis of eigenstates of the $\sigma_{z}$ Pauli matrix. The stochastic Lindblad equation for these couplings is

(2) $$\begin{array}{cc} d\rho & =\left(c_{+}\rho c_{+}^{†}-\frac{1}{2}\rho c_{+}^{†}c_{+}-\frac{1}{2}c_{+}^{†}c_{+}\rho \right)dt\\ \; & +\left(\rho c_{+}^{†}+c_{+}\rho -\mathrm{Tr}\left[\left(c_{+}+c_{+}^{†}\right)\rho \right]\rho \right)dW_{1}\\ \; & +\left(c_{-}\rho c_{-}^{†}-\frac{1}{2}\rho c_{-}^{†}c_{-}-\frac{1}{2}c_{-}^{†}c_{-}\rho \right)dt\\ \; & +\left(\rho c_{-}^{†}+c_{-}\rho -\mathrm{Tr}\left[\left(c_{-}+c_{-}^{†}\right)\rho \right]\rho \right)dW_{2}, \end{array}$$

where $dW_{1,2}$ are independent Wiener increments with $\langle dW_{1}\rangle =\langle dW_{2}\rangle =\langle dW_{1}dW_{2}\rangle =0$ and $\langle {\left(dW_{1}\right)}^{2}\rangle =\langle {\left(dW_{2}\right)}^{2}\rangle =dt$, where angled brackets denote stochastic averages [2,3,7]. Expressing ρ in terms of components of the coherence or Bloch vector ***r*** $=\left(r_{x},r_{y},r_{z}\right)\equiv \left(x,y,z\right)$, we write

(3) $$\rho =\frac{1}{2}\left(\begin{array}{cc} 1+z & x-iy\\ x+iy & 1-z \end{array}\right),$$

or $\rho =\frac{1}{2}\left(I+r\cdot \sigma \right)$ in terms of Pauli matrices $\sigma_{i}$. After some manipulation, we obtain three SDEs describing the dynamics of the system:

(4) $$\left(\begin{array}{c} dx\\ dy\\ dz \end{array}\right)=\left(\begin{array}{c} -x\\ -y\\ -2z \end{array}\right)dt+\left(\begin{array}{cc} 1-x^{2}-z & 1-x^{2}+z\\ -xy & -xy\\ x\left(1-z\right) & -x\left(1+z\right) \end{array}\right)\left(\begin{array}{c} dW_{1}\\ dW_{2} \end{array}\right),$$

which take the form

(5) $$dr_{i}=A_{i}\left(r\right)dt+\sum_{j}B_{ij}\left(r\right)dW_{j},$$

in terms of a vector A and matrix B.

**Figure 1:**
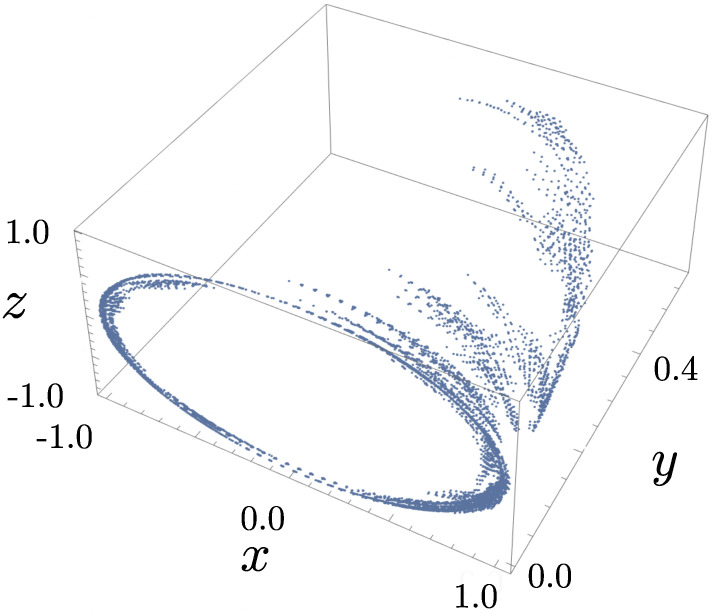
Example evolution of the coherence vector r=(x,y,z) governed by Equation (4). The trajectory was based on 10,000 steps with timestep $dt=10^{-3}$ and initiated at the point x=y=z=0.5 (an impure initial reduced density matrix). The coherence vector appears to be confined to an ellipsoid and to be driven towards a circle of unit radius in the *x*–*z* plane, corresponding to evolution towards purity.

**Figure 2:**
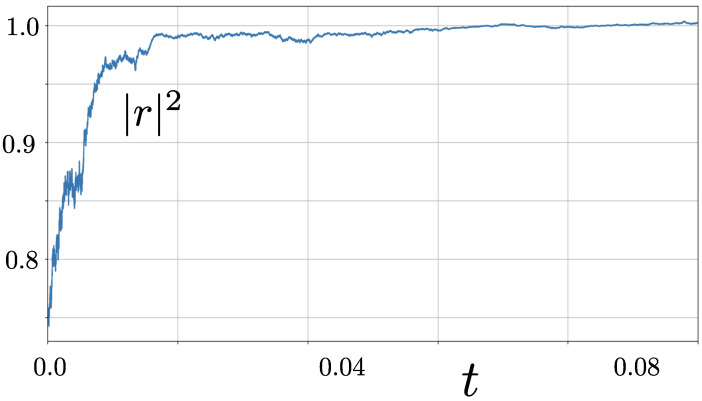
Evolution of the magnitude of the coherence vector r under the dynamics described by Equation (4). We see that ${\left|r\right|}^{2}$ is driven stochastically towards unity, which is synonymous with evolution towards purity, P=1. The trajectory was initiated at the point x=y=z=0.5 and constructed using a timestep of $dt=10^{-5}$.

The number of coordinates (three) is larger than the number of independent noise terms (two), suggesting that a description of the diffusive evolution exists involving only two coordinates. Indeed, an example of the evolution of r in its phase space in Figure 1 suggests the motion is constrained to lie on the surface of an ellipsoid that lies inside the Bloch sphere defined by |r|=r=1. The purity of the system $P=\mathrm{Tr}\rho^{2}=\frac{1}{2}\left(1+r^{2}\right)$ evolves according to

(6) $$dP=4(1-P)\left(1-x^{2}\right)dt+4x\left(1-P\right)\left(dW_{1}+dW_{2}\right),$$

suggesting that purification P→1 takes place as t→∞. This is demonstrated in Figure 2.

### 3.2. Stochastic Entropy Production

Stochastic entropy production is a measure of the irreversibility of an evolution governed by stochastic dynamics. It is associated with individual trajectories of a system and is defined as the logarithm of the probability of a ‘forward’ stochastic trajectory divided by the probability of a time-reversed or ‘backward’ trajectory driven by a time-reversed Hamiltonian applied after the forward evolution [1]. If these probabilities are equal then there is no statistical difference between forward and backward stochastic evolution and the statistical state of the system prior to the forward trajectory may be recovered. This means there is no irreversibility and no arrow of time. In many circumstances, however, the stochastic entropy production associated with an individual evolution is non-zero, and the second law of thermodynamics generally states that its average is non-negative.

The incremental stochastic entropy production is usually separated into system and environmental components as follows:

(7) $$d\Delta s_{\mathrm{tot}}=d\Delta s_{\mathrm{sys}}+d\Delta s_{\mathrm{env}},$$

with $d\Delta s_{\mathrm{sys}}=-d\ln p(r,t)$, where p(r,t) is the probability density function (pdf) characterising the stochastic dynamical variables and is governed by an appropriate Fokker–Planck equation. The average of the system component $d\Delta s_{\mathrm{sys}}$ over all possible trajectories is related to the incremental change in Gibbs entropy of the system, $d\langle \Delta s_{\mathrm{sys}}\rangle =dS_{G}$, to which boundary terms should be added in certain circumstances [2], as shown in Appendix C. We shall encounter a case where boundary contributions are important in Section 4.

An SDE for the evolution of the environmental component $d\Delta s_{\mathrm{env}}$ of stochastic entropy production is given in Appendix A. The derivation of this SDE is highly technical but emerges from considering the evolution of the logarithm of the ratio of probabilities referred at the start of this section as a consequence of the Markovian stochastic evolution of the system coordinates [13]. A significant problem in computing $d\Delta s_{\mathrm{env}}$ using this SDE is that the determinant of the diffusion matrix in the Fokker–Planck equation can be zero in some circumstances, making it impossible to define the inverse matrix needed in Equation (A4). This pathology, however, can be overcome, as we now demonstrate using procedures summarised in [7] and Appendix B.

### 3.3. Environmental Stochastic Entropy Production in x,z Coordinates

The confinement of the trajectory to an ellipsoid in Figure 1 is a consequence of the existence of a constant function of the motion

(8) $$f\left(x,y,z\right)=\frac{1}{y^{2}}(1-x^{2}-z^{2}),$$

which evolves by Itô’s lemma according to

(9) $$\begin{array}{cc} df & =\sum_{i}\frac{\partial f}{\partial r_{i}}dr_{i}+\sum_{i,j}\frac{\partial^{2}f}{\partial r_{i}\partial r_{j}}D_{ij}dt, \end{array}$$

where elements of the diffusion matrix are $D_{ij}=\frac{1}{2}\sum_{k}B_{ik}B_{jk}$ and the matrix B is defined in Equation (5). Using Equation (4), we obtain

(10) $$D=\left(\begin{array}{ccc} x^{4}-2x^{2}+z^{2}+1 & xy\left(x^{2}-1\right) & xz\left(x^{2}-2\right)\\ xy\left(x^{2}-1\right) & x^{2}y^{2} & x^{2}yz\\ xz\left(x^{2}-2\right) & x^{2}yz & x^{2}\left(z^{2}+1\right) \end{array}\right),$$

which is singular, as expected. By inserting this matrix into Equation (9), we find that df=0, demonstrating *f* to be a constant of the motion.

**Figure 3:**
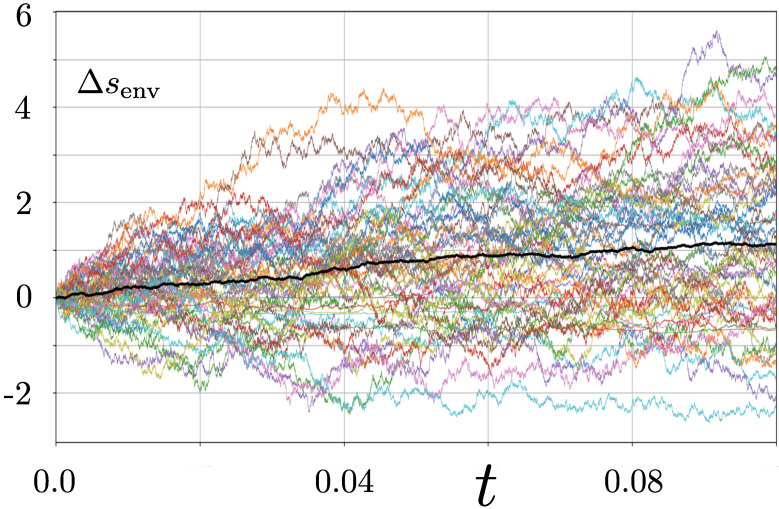
Environmental component of stochastic entropy production computed using Equation (A4) for 50 realisations of the dynamics described by Equation (11). The black line represents the ensemble mean. Each trajectory was initiated at the point x=z=0.5 and was generated using a timestep of $dt=10^{-5}$.

The constancy of *f* creates a situation characterised by restricted diffusion [7]. The dynamics in Equation (4) express diffusive evolution in an N=3 dimensional phase space driven by M=2 noise terms. Following the arguments summarised in Appendix B, we seek to identify the single $\left(N-M=1\right)$ eigenvector of D with an eigenvalue equal to zero, finding this to be $\alpha ={\left(\begin{array}{ccc} \frac{x}{z}, & \frac{1-x^{2}-z^{2}}{yz}, & 1 \end{array}\right)}^{T}$. The existence of such a ‘null’ eigenvector allows us to remove one of the SDEs from the dynamics. If we eliminate the evolution of coordinate *y*, the dynamics may be expressed as

(11) $$\left(\begin{array}{c} dx\\ dz \end{array}\right)=\left(\begin{array}{c} -x\\ -2z \end{array}\right)dt+\left(\begin{array}{cc} 1-x^{2}-z & 1-x^{2}+z\\ x\left(1-z\right) & -x\left(1+z\right) \end{array}\right)\left(\begin{array}{c} dW_{1}\\ dW_{2} \end{array}\right),$$

and the resulting reduced diffusion matrix is given by

(12) $$D_{\mathrm{red}}=\left(\begin{array}{cc} x^{4}-2x^{2}+z^{2}+1 & xz\left(x^{2}-2\right)\\ xz\left(x^{2}-2\right) & x^{2}\left(z^{2}+1\right) \end{array}\right),$$

which is non-singular. We can now compute trajectories for $d\Delta s_{\mathrm{env}}$ using Equation (A4) with Equation (11) and the reduced diffusion matrix (12). Figure 3 illustrates 50 realisations of the dynamics together with the average evolution.

The persistent average increase in $d\Delta s_{\mathrm{env}}$ with time seems to be a pathology: we might expect entropy production to cease as the system becomes thermalised. However, the dynamics considered here are incapable of achieving thermalisation, by which we mean stationarity of the reduced density matrix, in finite time. The purity approaches unity only as t→∞, according to Equation (6), and as this evolution continues, stochastic entropy production will continue. The situation is analogous to behaviour found in a two-level system undergoing measurement [3]. Thermalisation is only achieved asymptotically, and the production of stochastic entropy continues to characterise progress in this direction. This resolves the issue, but in the next section, we turn to a more puzzling matter.

## 4. Pathologies in Stochastic Entropy Production When Thermalising a Pure State

Consider the situation where purity has been established such that y=0 and $x^{2}=1-z^{2}$. The coherence vector then evolves randomly on a circle of unit radius in the *x*–*z* plane, on the Bloch sphere, and the stochastic dynamics and stochastic entropy production for further relaxation of the pure state towards a stationary state involve the evolution of a single coordinate *z*. We give this situation our attention in order to observe and then resolve an apparent pathology in the stochastic entropy production.

Inserting the limits for *x* and *y* into Equation (4) leads to an SDE,

(13) $$dz=-2zdt+{\left(2\left(1-z^{4}\right)\right)}^{1/2}dW,$$

describing the thermalisation of a pure state of the two-level system driven by the Lindblads $c_{\pm }$. Note that in the absence of a system Hamiltonian, the thermal state will be characterised by equal occupational probabilities of the two levels, each represented by coherence vectors at the north and south poles of the Bloch sphere, or z=±1. The mean value of *z* will tend asymptotically to zero as a consequence. Note that if the Lindblad operators were unequally weighted, for example, using scaled versions ${\left(1-\gamma /2\right)}^{1/2}c_{+}$ and ${\left(1+\gamma /2\right)}^{1/2}c_{-}$ where γ is a constant, the SDE would read

(14) $$dz=-\left(\gamma +2z\right)dt+{\left(2\left(1-z^{2}\right)\left(1+\gamma z+z^{2}\right)\right)}^{1/2}dW,$$

which has a stationary state with 〈z〉=−γ/2.

However, there are difficulties in computing the stochastic entropy production associated with the dynamics of Equation (13), which we now identify.

### 4.1. Environmental Component

Referring to Equation (13) and Appendix A, the environmental component of stochastic entropy production takes the form

(15) $$\begin{array}{ccc} d\Delta s_{\mathrm{env}} & = & \frac{A^{\mathrm{irr}}}{D}dz+\frac{\partial A^{\mathrm{irr}}}{\partial z}dt-\frac{1}{D}\frac{\partial D}{\partial z}dz\\ & & -\frac{A^{\mathrm{irr}}}{D}\frac{\partial D}{\partial z}dt-\frac{\partial^{2}D}{\partial z^{2}}dt+\frac{1}{D}{\left(\frac{\partial D}{\partial z}\right)}^{2}dt,\; \end{array}$$

since $A^{\mathrm{irr}}=-2z$, $A^{\mathrm{rev}}=0$ and diffusion coefficient $D=1-z^{4}$. Inserting $dD/dz=-4z^{3}$ and $d^{2}D/dz^{2}=-12z^{2}$, we obtain

(16) $$\begin{array}{cc} d\Delta s_{\mathrm{env}} & =2z\frac{\left(2z^{2}-1\right)}{{\left(1-z^{4}\right)}^{1/2}}2^{1/2}dW\\ \; & +\frac{2}{1-z^{4}}\left(-1-7z^{4}+8z^{2}+2z^{6}\right)dt, \end{array}$$

which has singularities at z=±1. This potentially creates difficulties in evaluating $d\Delta s_{\mathrm{env}}$ numerically.

### 4.2. System Component

**Figure 4:**
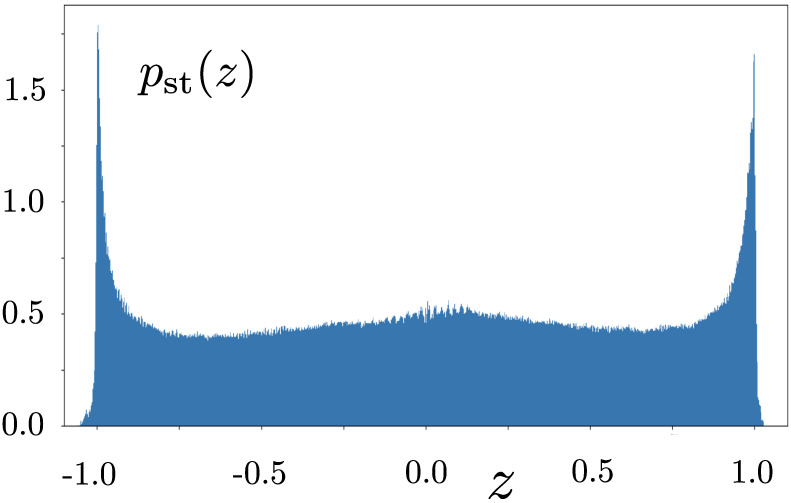
Histogram illustrating the stationary pdf of *z* generated from a solution to Equation (4) using $10^{7}$ timesteps of size $dt=10^{-3}$. The trajectory was initiated at the point x=y=z=0.5 and the bin size is $10^{-4}$. Non-zero values for |z|>1 are numerical artefacts.

These difficulties become apparent when we compute the system component of stochastic entropy production. Consider the histogram of values of *z* in Figure 4, obtained from a single trajectory generated using the set of SDEs in Equation (4). The dynamics clearly allow *z* to take values ±1, suggesting that the potential singularities in the calculation of the environmental component of stochastic entropy production identified in Equation (16) might be encountered.

We can derive the stationary pdf analytically to illustrate this further. The Fokker–Planck equation associated with Equation (13) is

(17) $$\frac{\partial p(z,t)}{\partial t}=-\frac{\partial J}{\partial z},$$

where the probability current is

(18) $$J=-2zp-\frac{\partial }{\partial z}\left(\left(1-z^{4}\right)p\right).$$

The stationary pdf in −1≤z≤1 is therefore given by

(19) $$p_{\mathrm{st}}(z)\propto \frac{1}{{\left(1-z^{4}\right)}^{1/2}}\frac{1}{1+z^{2}},$$

which possesses singularities at z=±1, consistent with the numerical results in Figure 4.

Note that the stationary expectation value $\int_{-1}^{1}zp_{\mathrm{st}}(z)dz=0$ is compatible with the asymptotic solution to the evolution d〈z〉=−2〈z〉dt obtained by averaging the SDE (13) over the noise and employing 〈dW〉=0, where angled brackets denote stochastic averages. In Appendix C, we show, however, that this result need not hold if the product of diffusion coefficient and pdf at the boundaries does not vanish; an extra term makes a contribution such that

(20) $$d\langle z\rangle =-2\langle z\rangle dt-{\left[Dp\right]}_{z=-1}^{z=1}dt.$$

In this case, however, we have $Dp_{\mathrm{st}}\propto {\left(1-z^{4}\right)}^{1/2}/\left(1+z^{2}\right)$, which vanishes at z=±1. Assuming, not unreasonably, that this feature is preserved when the system is nonstationary, the usual outcome d〈z〉=−2〈z〉dt of the stochastic averaging of Equation (13) emerges.

More significantly, boundary terms also appear in the evolution of the mean system component of stochastic entropy production, as already alluded to in Section 3.2. According to Equation (A24), the relationship between this quantity and the change in Gibbs entropy of the system $S_{G}$ is

(21) $$\begin{array}{cc} d\langle \Delta s_{\mathrm{sys}}\rangle & =\frac{dS_{G}\left(t\right)}{dt}dt-{\left[J\ln p\right]}_{z=-1}^{z=1}dt-{\left[D\frac{\partial p}{\partial z}\right]}_{z=-1}^{z=1}dt\\ - & {\left[D{\left(\frac{\partial \ln p}{\partial z}\right)}^{2}P\left(\Delta s_{\mathrm{sys}},t\right)\right]}_{\Delta s_{\mathrm{sys}}^{\min}}^{\Delta s_{\mathrm{sys}}^{\max}}dt, \end{array}$$

where *P* is the pdf of the quantity $\Delta s_{\mathrm{sys}}$. The second term on the right-hand side vanishes since J=0 at the boundaries and the fourth can be simplified using $P\left(\Delta s_{\mathrm{sys}},t\right)d\Delta s_{\mathrm{sys}}=pdz$. In the stationary state, both the third and fourth terms depend on the expression

(22) $$\begin{array}{cc} D\frac{dp_{\mathrm{st}}}{dz} & \propto \left(1-z^{4}\right)(2z^{3}{\left(1-z^{4}\right)}^{-3/2}{\left(1+z^{2}\right)}^{-1}\\ \; & -2z{\left(1-z^{4}\right)}^{-1/2}{\left(1+z^{2}\right)}^{-2}), \end{array}$$

which is singular at z=±1. We therefore expect difficulties to arise in computing the system stochastic entropy production, in addition to those already encountered in the evaluation of $\Delta s_{\mathrm{env}}$. But are these genuine problems?

### 4.3. Removal of Pathological Behaviour

The pathologies encountered in the calculation of $d\Delta s_{\mathrm{tot}}$ may be removed by a simple change in the coordinate frame. Defining $\theta =\cos^{-1}z$ and using Itô’s lemma, we find that the dynamics of the pure state may also be described by the SDE

(23) $$d\theta =\frac{1}{2}\sin2\theta dt+{\left(2\left(1+\cos^{2}\theta \right)\right)}^{1/2}dW,$$

instead of Equation (13) for *z*. The diffusion coefficient is $D=1+\cos^{2}\theta$. The stationary pdf in θ is

(24) $${\hat{p}}_{\mathrm{st}}\left(\theta \right)\propto {\left(1+\cos^{2}\theta \right)}^{-3/2},$$

and so $D{\hat{p}}_{\mathrm{st}}\propto {\left(1+\cos^{2}\theta \right)}^{-1/2}$ such that the boundary term ${\left[D{\hat{p}}_{\mathrm{st}}\right]}_{\theta =0}^{\theta =\pi }$ vanishes. As a consequence, and as before, the averaging of the dynamics in Equation (23) proceeds along usual lines, giving $d\langle \theta \rangle =\frac{1}{2}\langle \sin2\theta \rangle dt$.

Furthermore, we have

(25) $$D\frac{d{\hat{p}}_{\mathrm{st}}}{d\theta }\propto \frac{\sin2\theta }{1+\cos^{2}\theta },$$

which is zero at θ=0 and π. As a consequence, the boundary terms in the evolution of mean system stochastic entropy production in the stationary state vanish. It is therefore not unreasonable to expect that even in a nonstationary situation, the evolution should satisfy

(26) $$\begin{array}{cc} d\langle \Delta s_{\mathrm{sys}}\rangle & =\frac{dS_{G}(t)}{dt}dt, \end{array}$$

again in line with what is typically assumed.

Finally, the evolution of environmental stochastic entropy production can be identified using the SDE for θ:

(27) $$d\Delta s_{\mathrm{env}}=\frac{6+18\cos2\theta +3\sin^{2}2\theta }{4\left(1+\cos^{2}\theta \right)}dt+\frac{3\sin2\theta }{\sqrt{2}(1+\cos^{2}\theta )}dW,$$

in which there are again no singularities at the boundaries. We conclude that all the pathologies disappear when an appropriate coordinate (θ rather than *z*) is used to describe the dynamics and thermodynamics.

## 5. Conclusions

The main purpose in presenting this study is to identify interpretational and mathematical issues concerning stochastic entropy production in a model of the thermalisation of a two-level quantum system, and then to resolve them. The treatment of open system dynamics as a continuous Brownian motion of a physical density matrix, a quantum state diffusion, is perhaps unfamiliar, and this is particularly so for the consideration of stochastic entropy production as the outcome of an Itô process. The mean value of stochastic entropy production is a measure of the change in subjective uncertainty in the adopted state of the world given the employment of a coarse-grained model of its evolution. We need to address any apparent pathologies in the calculation of stochastic entropy production that might cast doubt on its use in this role.

Our conclusions are as follows. Thermalisation of a two-level system can be achieved through coupling to a coarse-grained environment by way of raising and lowering operators. Such an interaction is not the same as quantum measurement, but the model we use nevertheless purifies the reduced density matrix asymptotically in time, and this brings about a persistent production of stochastic entropy. This is not a pathology. The model of thermal interactions we use disentangles a system from its environment, creating greater subjective uncertainty in the state of the world as a less correlated system behaviour emerges. Note that an impure or mixed reduced density matrix, such as the Gibbs state often used to describe a thermalised system, may be interpreted as the *average* of an ensemble of reduced density matrices. A mixed reduced density matrix does not necessarily imply entanglement.

Furthermore, if the two-level system is thermalised starting from a disentangled or pure state, the stochastic entropy production can appear to be pathological in both the environmental and system components. It turns out, however, that such a conclusion is an artefact of a particular choice of coordinate system and a simple transformation of variables can remove the mathematical difficulties. Stochastic entropy production should not depend on choice of coordinates, but some are clearly more suitable than others. We note as a corollary that the separate system and environmental components of stochastic entropy production *are* coordinate frame-dependent. Gibbs entropy, for example, expresses uncertainty of adopted system state in a particular coordinate phase space.

In conclusion, we have presented arguments to support the use of stochastic entropy production in open quantum system dynamics in a manner similar to its employment in classical situations. Its role and the implications are just the same and, as we have suggested, just as profound.

## Data Availability

All relevant data are contained within the article.

## Appendix A. Stochastic Entropy Production

We consider a set of coordinates $x\equiv (x_{1},x_{2},\cdots )$ that specify the configuration of a system, and model their evolution using Itô processes:

(A1) $$dx_{i}=A_{i}\left(x\right)dt+\sum_{j}B_{ij}\left(x\right)dW_{j},$$

where the $dW_{j}$ are independent Wiener increments. The Fokker–Planck equation for the pdf p(x,t) is

(A2) $$\frac{\partial p}{\partial t}=-\sum_{i}\frac{\partial }{\partial x_{i}}\left(A_{i}p\right)+\frac{\partial }{\partial x_{i}\partial x_{j}}\left(D_{ij}p\right),$$

in terms of a diffusion matrix $D\left(x\right)=\frac{1}{2}B\left(x\right)B{\left(x\right)}^{T}$. The stochastic entropy production of the system and its environment associated with the stochastic dynamics is given by

(A3) $$d\Delta s_{\mathrm{tot}}=d\Delta s_{\mathrm{sys}}+d\Delta s_{\mathrm{env}},$$

with $d\Delta s_{\mathrm{sys}}=-d\ln p(x,t)$. If we define $A_{i}^{\mathrm{irr}}\left(x\right)=\frac{1}{2}\left[A_{i}(x)+\varepsilon_{i}A_{i}(\varepsilon x)\right]$ and $A_{i}^{\mathrm{rev}}\left(x\right)=\frac{1}{2}\left[A_{i}(x)-\varepsilon_{i}A_{i}(\varepsilon x)\right]$ [6], where $\varepsilon_{i}=1$ for variables $x_{i}$ with even parity under time-reversal symmetry (for example, position) and $\varepsilon_{i}=-1$ for variables with odd parity (for example, velocity), with εx representing $\left(\varepsilon_{1}x_{1},\varepsilon_{2}x_{2},\cdots \right)$, it may be shown that [13]

(A4) $$\begin{array}{cc} & d\Delta s_{\mathrm{env}}=-\sum_{i}\frac{\partial A_{i}^{\mathrm{rev}}}{\partial x_{i}}dt+\sum_{i,j}\{D_{ij}^{-1}A_{i}^{\mathrm{irr}}dx_{j}\\ \; & -D_{ij}^{-1}\sum_{m}\frac{\partial D_{im}}{\partial x_{m}}dx_{j}-D_{ij}^{-1}A_{i}^{\mathrm{rev}}A_{j}^{\mathrm{irr}}dt\\ \; & +D_{ij}^{-1}A_{i}^{\mathrm{rev}}\sum_{n}\frac{\partial D_{jn}}{\partial x_{n}}dt+\sum_{k}[D_{ik}\frac{\partial }{\partial x_{k}}\left(D_{ij}^{-1}A_{j}^{\mathrm{irr}}\right)\\ \; & -D_{ik}\frac{\partial }{\partial x_{k}}\left(D_{ij}^{-1}\sum_{n}\frac{\partial D_{jn}}{\partial x_{n}}\right)]dt\}. \end{array}$$

The derivation is highly technical, unfortunately. For the dynamics considered in the main text, all coordinates are of even parity.

## Appendix B. Dimensional Reduction of a Singular Diffusion Matrix

Consider a system evolving stochastically in an *N* dimensional phase space under the influence of *M* noise terms such that the B matrix in Equation (A1) is N×M and the diffusion matrix D in the Fokker–Planck Equation (A2) is N×N. The phase space is described by a set of *N* coordinates $\left\{x_{1},x_{2},\ldots ,x_{N}\right\}$, and we conjecture that there exist L=N−M functions of these coordinates that are invariant under the dynamics, or more generally, evolve without noise. These allow us to remove *L* coordinates from the *N* dimensional phase space and consider evolution in a reduced *M* dimensional phase space. We call the *M* coordinates $\left(\left\{x_{m}\right\}\right)$ of the reduced phase space *dynamical variables* and the remaining *L* coordinates $\left(\left\{x_{l}\right\}\right)$ *spectator variables*. The *L* constraints allow us to write the spectator variables as functions of the dynamical variables: $\left\{x_{l}\left(\left\{x_{m}\right\}\right)\right\}$. The diffusion matrix in the reduced phase space will be M×M and non-singular, allowing us to use Equation (A4) to compute the stochastic entropy production associated with the evolution [7].

If f(x) is a constant of the motion, we can regard ∇f as a ‘null’ eigenvector of D with zero eigenvalue. Furthermore, since the infinitesimal displacement dx lies on the hypersurface of *f* to which the evolution is confined, we also have ∇f·dx=0. Combining these, we write

(A5) $$\sum_{m=1}^{M}\alpha_{km}dx_{m}+\sum_{l=1}^{L}\alpha_{kl}dx_{l}=0,$$

where $\alpha_{km}$ and $\alpha_{kl}$ are the dynamical and spectator components of the *k*th null eigenvector of D. The $\alpha_{km}$ are elements of a rectangular L×M matrix $Q_{km}$ and the $\alpha_{kl}$ are elements of a square L×L matrix $P_{kl}$ so that $P_{kl}dx_{l}=-Q_{km}dx_{m}$ and

(A6) $$\begin{array}{cc} dx_{l} & =-P_{lk}^{-1}Q_{km}dx_{m}=R_{lm}dx_{m}, \end{array}$$

where summations over repeated indices are implied and $R_{lm}$ is a component of the L×M matrix R. Next, we write

(A7) $$dD_{ij}=\sum_{m=1}^{M}\frac{\partial D_{ij}}{\partial x_{m}}dx_{m}+\sum_{l=1}^{L}\frac{\partial D_{ij}}{\partial x_{l}}dx_{l},$$

and substituting from Equation (A6), we obtain the derivative of $D_{ij}$ with respect to the dynamical coordinate $x_{m}$ in the form

(A8) $$\frac{dD_{ij}}{dx_{m}}=\frac{\partial D_{ij}}{\partial x_{m}}+\sum_{l}\frac{\partial D_{ij}}{\partial x_{l}}R_{lm}.$$

Equation (A4) can be used to compute stochastic entropy production based on the dynamics of the *M* dynamical variables with derivatives modified according to Equation (A8). Additional discussion of the approach is given elsewhere [7].

## Appendix C. System Component of Mean Stochastic Entropy Production

It is typically assumed that the average of the $d\Delta s_{\mathrm{sys}}=-d\ln p\left(x,t\right)$ term in the expression for the incremental stochastic entropy production corresponds to the change in system Gibbs entropy, but this is not necessarily the case, as we now show.

Consider the SDE for a function g(x) of the stochastic variables x:

(A9) $$dg=\hat{A}(x)dt+\hat{B}(x)dW,$$

with pdf $\hat{p}(g,t)$ satisfying

(A10) $$\frac{\partial \hat{p}(g,t)}{\partial t}=-\frac{\partial \hat{J}}{\partial g},$$

in terms of a probability current $\hat{J}$ given by

(A11) $$\hat{J}=\hat{A}\hat{p}-\frac{1}{2}\frac{\partial }{\partial g}\left({\hat{B}}^{2}\hat{p}\right).$$

Defining a stochastic average of *g* as ${\langle g\rangle }_{t}=\int g\hat{p}(g,t)dg$, we consider the difference d〈g〉 between ${\langle g\rangle }_{t+dt}$ and ${\langle g\rangle }_{t}$:

(A12) $$\begin{array}{cc} d\langle g\rangle & =\int g\left(\hat{p}\left(g,t+dt\right)-\hat{p}\left(g,t\right)\right)dg=\int g\frac{\partial \hat{p}}{\partial t}dgdt\\ \; & =-\int g\frac{\partial \hat{J}}{\partial g}dgdt=-\left[g\hat{J}\right]dt+\int \hat{J}\;dgdt, \end{array}$$

where the square brackets indicate boundary terms. But since $\hat{J}=0$ at the boundaries in the absence of probability current sources and sinks, the first term vanishes and so

(A13) $$\begin{array}{cc} d\langle g\rangle & =\int \left(\hat{A}\hat{p}-\frac{1}{2}\frac{\partial }{\partial g}\left({\hat{B}}^{2}\hat{p}\right)\right)dgdt\\ \; & ={\langle \hat{A}\rangle }_{t}dt-\frac{1}{2}\left[{\hat{B}}^{2}\hat{p}\right]dt, \end{array}$$

where another boundary term has appeared. Note that the average in the first term on the right-hand side can be written in two forms: ${\langle \hat{A}\rangle }_{t}=\int \hat{A}(x)\hat{p}(g,t)dg=\int \hat{A}(x)p(x,t)dx$. The second version involves the pdf p(x,t) over the stochastic variables x, as specified by the Fokker–Planck equation

(A14) $$\frac{\partial p}{\partial t}=-\sum_{j}\frac{\partial J_{j}}{\partial x_{j}},$$

associated with the dynamics $dx_{i}=A_{i}dt+\sum_{j}B_{ij}dW_{j}$ and with probability current

(A15) $$J_{j}=A_{j}p-\sum_{i}\frac{\partial }{\partial x_{i}}\left(D_{ij}p\right),$$

where $D_{ij}=\frac{1}{2}\sum_{k}B_{ik}B_{jk}$.

The first term on the right-hand side of Equation (A13) is perhaps expected to be the only contribution after averaging Equation (A9), but it is augmented by a term that depends on the values of ${\hat{B}}^{2}\hat{p}$ at the boundaries of the domain of *g*. This extra term could vanish but it is not guaranteed to do so.

Consider, for example, a single stochastic variable evolving as dx=adt+bdW: the stochastic average satisfies d〈x〉/dt=〈a〉−[Dp] where $D=b^{2}/2$. In many cases, the pdf vanishes at the boundaries, or for periodic boundary conditions the contributions might cancel. It is only in certain cases that further attention is required. An explicit case involves a(x)=x and $b\left(x\right)=x^{2}$ in the domain 0≤x≤∞. The stationary pdf is $p_{\mathrm{st}}(x)=4\pi^{-1/2}x^{-4}\exp(-1/x^{2})$ and the diffusion coefficient is $D=x^{4}/2.$ Hence, $Dp_{\mathrm{st}}$ vanishes at x=0 but is equal to $2/\pi^{1/2}$ at x=∞. We should hence model the evolution of 〈x〉 according to $d\langle x\rangle /dt=\langle x\rangle -2/\pi^{1/2}$, which is consistent with a non-zero mean of *x* as t→∞, as suggested by the stationary pdf. The boundary term is crucial in achieving this.

Let us now analyse the evolution of the system stochastic entropy production, $g(x)=\Delta s_{\mathrm{sys}}=-\ln p(x,t)$. We write

(A16) $$\begin{array}{cc} d\Delta s_{\mathrm{sys}} & =-\frac{\partial \ln p}{\partial t}dt-\sum_{j}\frac{\partial \ln p}{\partial x_{j}}dx_{j}-\sum_{ij}D_{ij}\frac{\partial^{2}\ln p}{\partial x_{i}\partial x_{j}}dt\\ \; & =\hat{A}dt+\sum_{ij}{\hat{B}}_{ij}dW_{j}=\hat{A}dt+\hat{B}dW, \end{array}$$

with

(A17) $$\hat{A}=-\frac{\partial \ln p}{\partial t}-\sum_{j}\frac{\partial \ln p}{\partial x_{j}}A_{j}-\sum_{ij}D_{ij}\frac{\partial^{2}\ln p}{\partial x_{i}\partial x_{j}},$$

and

(A18) $${\hat{B}}_{ij}=-\frac{\partial \ln p}{\partial x_{i}}B_{ij},$$

such that

(A19) $$\begin{array}{cc} \hat{B} & ={\left(\sum_{ijk}{\hat{B}}_{kj}{\hat{B}}_{ij}\right)}^{1/2}={\left(\sum_{ijk}\frac{\partial \ln p}{\partial x_{k}}\frac{\partial \ln p}{\partial x_{i}}B_{kj}B_{ij}\right)}^{1/2}\\ \; & ={\left(2\sum_{ik}\frac{\partial \ln p}{\partial x_{k}}\frac{\partial \ln p}{\partial x_{i}}D_{ki}\right)}^{1/2}. \end{array}$$

The mean system stochastic entropy production therefore satisfies

(A20) $$d\langle \Delta s_{\mathrm{sys}}\rangle ={\langle \hat{A}\rangle }_{t}dt-\frac{1}{2}\left[{\hat{B}}^{2}P\left(\Delta s_{\mathrm{sys}},t\right)\right]dt,$$

where *P* is the pdf of $\Delta s_{\mathrm{sys}}$. $\hat{B}$ is a function of the stochastic variables x and the second term is evaluated at the boundary values of $\Delta s_{\mathrm{sys}}$ using the appropriate values of x.

We further develop the first term on the right-hand side of Equation (A20), writing

(A21) $${\langle \hat{A}\rangle }_{t}=\int \hat{A}\left(x\left(\Delta s_{\mathrm{sys}}\right)\right)P\left(\Delta s_{\mathrm{sys}},t\right)d\Delta s_{\mathrm{sys}},$$

and noting that for a given $\Delta s_{\mathrm{sys}}$, there are potentially several corresponding values of x that contribute to the integrand. It is more convenient to cast the integral in terms of x instead of $\Delta s_{\mathrm{sys}}$, namely to write ${\langle \hat{A}\rangle }_{t}=\int \hat{A}\left(x\right)p\left(x,t\right)dx$ and employing integration limits for x instead of $\Delta s_{\mathrm{sys}}$. Continuing the development, we find that

(A22) $$\begin{array}{cc} {\langle \hat{A}\rangle }_{t} & =\int \left(-\frac{\partial \ln p}{\partial t}-\sum_{j}\frac{\partial \ln p}{\partial x_{j}}A_{j}-\sum_{ij}D_{ij}\frac{\partial^{2}\ln p}{\partial x_{i}\partial x_{j}}\right)p\;dx\\ = & \int \left(-\frac{\partial \ln p}{\partial t}p-\sum_{j}\left(A_{j}p-\frac{\partial \left(D_{ij}p\right)}{\partial x_{i}}\right)\frac{\partial \ln p}{\partial x_{j}}\right)dx\\ \; & -\sum_{ij}\left[D_{ij}\frac{\partial \ln p}{\partial x_{j}}p\right]\\ = & \int \left(-\frac{\partial \ln p}{\partial t}p+\sum_{j}\frac{\partial J_{j}}{\partial x_{j}}\ln p\right)dx\\ \; & -\sum_{j}\left[J_{j}\ln p\right]-\sum_{ij}\left[D_{ij}\frac{\partial p}{\partial x_{j}}\right], \end{array}$$

reducing to

(A23) $$\begin{array}{cc} {\langle \hat{A}\rangle }_{t} & =\int \left(-\frac{\partial \ln p}{\partial t}p-\frac{\partial p}{\partial t}\ln p\right)dx\\ \; & -\sum_{j}\left[J_{j}\ln p\right]-\sum_{ij}\left[D_{ij}\frac{\partial p}{\partial x_{j}}\right]\\ = & -\frac{d}{dt}\int p\ln p\;dx-\sum_{j}\left[J_{j}\ln p\right]-\sum_{ij}\left[D_{ij}\frac{\partial p}{\partial x_{j}}\right]. \end{array}$$

Combining Equations (A20) and (A23), and inserting the Gibbs entropy $S_{G}(t)=-\int p\left(x,t\right)$lnp(x,t)dx, we arrive at

(A24) $$\begin{array}{cc} d\langle \Delta s_{\mathrm{sys}}\rangle & =\frac{dS_{G}(t)}{dt}dt-\sum_{j}\left[J_{j}\ln p\right]dt\\ \; & -\sum_{ij}\left[D_{ij}\frac{\partial p}{\partial x_{j}}\right]dt-\frac{1}{2}\left[{\hat{B}}^{2}P\left(\Delta s_{\mathrm{sys}},t\right)\right]dt, \end{array}$$

which is equivalent to Equation (21). The increment in mean system stochastic entropy production corresponds in part to the increment in Gibbs entropy $dS_{G}=\frac{dS_{G}}{dt}dt$, but three boundary terms also contribute, with the first two involving the edges of the phase space of the x coordinates, and the third involving the edges of the range of $\Delta s_{\mathrm{sys}}$.
